# Routing and Scheduling in Time-Sensitive Networking by Evolutionary Algorithms

**DOI:** 10.3390/biomimetics10050333

**Published:** 2025-05-20

**Authors:** Zengkai Wang, Weizhi Liao, Xiaoyun Xia, Zijia Wang, Yaolong Duan

**Affiliations:** 1 School of Artificial Intelligence, Jiaxing University, Jiaxing 314001, China; wangzengkai@zjxu.edu.cn (Z.W.); liaowz@zjxu.edu.cn (W.L.); 2 Technology Research and Development Centre, Xuelong Group Co., Ltd., Ningbo 315899, China; dyl7981@163.com; 3 School of Computer Science and Cyber Engineering, Guangzhou University, Guangzhou 510006, China

**Keywords:** Time-Sensitive Networking, routing and scheduling, evolutionary algorithm, differential evolution algorithm

## Abstract

Routing and scheduling in Time-Sensitive Networking (TSN) is an NP-hard problem. In this paper, we propose a novel routing and scheduling approach for TSN based on evolutionary algorithm. Specifically, we introduce a flow grouping method that leverages the greatest common divisor to optimize flow aggregation. On this basis, we develop a flow routing strategy that employs a genetic algorithm, where the evaluation function considers not only flow combinability but also path length and network load. By exploiting the non-combinable properties of flows, we effectively reduce the search space for the genetic algorithm. Furthermore, we design a scheduling method based on differential evolution algorithms tailored to TSN’s requirements of zero jitter and no frame loss. We propose a gene coding method and rigorously prove its correctness, which significantly reduces the search space of the differential evolution algorithm. The experimental results demonstrate that our approach enables more flows to traverse along the shortest path compared to both k-shortest path methods and integer linear programming approaches, while achieving a faster execution time in large-scale scheduling scenarios.

## 1. Introduction

With the rapid advancement of intelligent driving vehicle networks, the industrial Internet, and 5G communication technologies, an increasing number of data acquisition nodes and computing nodes have complicated network design and imposed significant data loads on communication links. Although the traditional Ethernet meets high bandwidth requirements and facilitates seamless connectivity among various specialized devices, it lacks essential real-time functionalities such as low jitter, minimal network latency, and efficient bandwidth allocation [1]. To address these limitations, the IEEE 802.1 standards have introduced a Time-Sensitive Networking (TSN) [2] solution that combines the cost-effectiveness and high-bandwidth capabilities of Ethernet with high-quality data transmission. Consequently, TSN-related technologies have garnered significant attention from both academia and industry. However, practical analysis and computation in TSN systems can become complex due to diverse network architectures and varying Quality of Service (QoS) requirements for different data flows. Studies indicate that routing and scheduling challenges within TSN are classified as NP-complete problems [3].

While most research has focused on addressing traffic scheduling using methods such as Satisfiability Modulo Theories (SMT) [4] and Integer Linear Programming (ILP) [5,6], these approaches often suffer from long solution times, limiting their practical applicability and scalability. In contrast, heuristic algorithms offer inherent scalability for solving routing and scheduling problems, leading to increased attention from researchers exploring TSN-based methods. In this paper, we focus on the routing and scheduling problem of TSN using evolutionary algorithms. A time-sensitive network routing and scheduling optimization scheme that deeply integrates genetic algorithm (GA) and differential evolution (DE) algorithms is proposed. Both evolutionary computing methods are inspired by the bionic simulation of biological evolution mechanisms. Genetic algorithms encode feasible routes as chromosomes, simulating the “survival of the fittest” principle in nature, and achieve iterative solution optimization through operations such as selection, crossover, and mutation. Differential evolution algorithms leverage the intelligent behavior of differentiated collaboration among biological populations, enhancing global search capabilities via vector differential perturbation. The synergy between the two algorithms mirrors the phenomenon of species coevolution in nature. Specifically, the genetic algorithm focuses on generating high-quality routing schemes, while the differential evolution algorithm refines the scheduling strategy, ultimately forming a bionic intelligent optimization framework. This approach not only demonstrates the robust adaptability of biological evolution principles in engineering optimization but also offers a novel bionic solution for complex constraint problems, such as those encountered in time-sensitive networks.

From the perspective of bionics, this method effectively translates the evolutionary intelligence of nature into engineering optimization tools. The fitness function in genetic algorithms mimics environmental selection pressure, the population cooperation mechanism in differential evolution replicates biological swarm intelligence, and the treatment of constraint conditions parallels the survival competition principle within an ecosystem. This bionic optimization strategy not only ensures the robustness of the algorithm in complex network environments but also, its hierarchical evolution architecture demonstrates self-organizing characteristics akin to those observed in biological systems. The research findings have comprehensively validated the practical significance of bionic intelligence theory in industrial communication systems, offering a critical methodological reference for the optimization of future intelligent networks.

The main contributions of our work are as follows.

(1) We proposed an innovative approach for route selection in TSN using a genetic algorithm for each flow. A fitness function that incorporates multiple factors including flow combinability, route length, and network load is formulated to identify routes that enhance efficient implementation of scheduling. To reduce the search space of the GA, we developed a method to eliminate infeasible routes by leveraging flow combinability analysis.
(2) An efficient method for finding a feasible scheduling solution for TSN based on differential evolution algorithm was developed. We proposed a straightforward and effective encoding scheme designed to substantially reduce the search space of the algorithm. Furthermore, we employ the differential evolution algorithm to tackle the feasible scheduling problem in TSN utilizing the number of constraints that must be satisfied by a feasible schedule as our objective function.

The rest of this paper is organized as follows. Section 2 provides a comprehensive review of the existing literature on the routing and scheduling of TSN. The route selection method based on the genetic algorithm is presented in Section 3. Section 4 details the scheduling approach for TSN that employs the differential evolution algorithm. The simulation results are analyzed in Section 5. Finally, Section 6 summarizes the findings of this study and outlines directions for future research.

## 2. Related Work

Real-time scheduling for TSN aims to determine a feasible network-wide configuration, including routing paths and schedules, which meets the end-to-end timing requirements of a given set of flows. In this section, we review related work on real-time scheduling of TSN. Existing work on time-sensitive network scheduling can be categorized into three distinct groups. The first category includes fixed route scheduling methods, which concentrate on determining the optimal routes for each flow to improve the scheduling success rate. The second category covers joint routing and scheduling (JRS) approaches, which aim to establish an integrated framework combining routing and scheduling while proposing solution strategies to tackle these associated challenges. The third category involves scheduling techniques for pre-determined routes, emphasizing the design of effective scheduling mechanisms to ensure flow schedulability once the routes are determined. We refer to these three categories as routing problems, joint routing and scheduling problems, and scheduling problems, respectively.

### 2.1. Routing

Greedy algorithms are widely employed to solve routing problems in TSN. By incorporating network performance metrics, researchers have developed various greedy algorithms to identify optimal route for each flow. For instance, A. Alnajim et al. proposed an incremental QoS-aware path selection algorithm that leverages QoS measurement to route TSN flows [7]. Shih-Hung Chang et al. proposed a real-time routing scheduler (RTRS) which determines both the shortest path and the path with the worst-case end-to-end delay (WCED) between source and destination nodes [8]. M. A. Ojewale et al. proposed selecting routes based on optimal load distribution among all valid paths [9]. Kai Huang et al. proposed a period-aware routing algorithm to alleviate scheduling bottlenecks, thereby accommodating a greater number of flows [10]. Despite their effectiveness, greedy algorithms suffer from an excessively large search space, leading to high time complexity. To address this issue, researchers have enhanced greedy algorithms through methods such as the K-shortest path approach [11] and the improved Dijkstra algorithm [12]. Additionally, heuristic search-based routing strategies have been proposed to effectively reduce the search space [13,14,15]. Moreover, some researchers have explored route selection methodologies using linear programming [16] and reinforcement learning [17,18], although these approaches demand substantial computational resources and runtime.

### 2.2. Joint Routing and Scheduling

The joint routing and scheduling approach offers the advantage of enabling integrated decision making for both the routing and scheduling of each flow. This integration enhances network resources utilization and improves schedulability. Integer Linear Programming and Satisfiability Modulus Theory can be employed to address the JRS problem, thereby minimizing the occurrences where the scheduling algorithm fails to find feasible solutions. The JRS problem can be formulated as an ILP model and solved using CPLEX and SMT solvers [19,20,21,22,23]. However, these methods are limited to small-scale problems due to their computational complexity, which increases significantly with the size of the problem. To address the challenges associated with large-scale JRS problems, researchers have explored alternative approaches. Xiaolong Wang et al. developed a greedy algorithm for JRS that incorporates load balancing [24]. Hao Yu et al. proposed a deep reinforcement learning-based deterministic flow scheduler to handle deterministic flow routing and scheduling [25]. N. Reusch et al. developed a novel way that integrates a simulated annealing metaheuristic with an ASAP list scheduling technique [26]. Jie Jia et al. proposed the two-level iterative JOORS algorithm, which is based on the differential evolution algorithm, to determine the injection time offset and routing strategies that maximize the scheduling success rate [27]. M. Pahlevan and R. Obermaisser et al. presented a genetic algorithm-based method for solving the JRS problem [28].

### 2.3. Scheduling

For the fixed route scheduling method, designing an efficient scheduling algorithm after the route has been computed is a critical issue. Many SMT and ILP formulations have been presented to determine a communication schedule based on precomputed routes [29,30,31]. A.A. Atallah et al. introduced a novel DoC-aware flow partitioning approach aimed at enhancing the success rate of iterated ILP-based scheduling [32]. However, due to the substantial computational demands associated with linear programming, researchers have increasingly focused on heuristic algorithms for scheduling methods. For instance, A. Berisa et al. proposed an AVB-aware heuristic scheduling algorithm which ensures the timely delivery of flows while optimizing the schedulability of AVB traffic [33]. M. Vlk et al. developed an Efficient Probing Instructed by Conflicts (EPIC)algorithm, which operates independently of third-party solvers [34]. F. Dürr et al. presented a Tabu search algorithm for efficient schedule computation and schedule compression technique to minimize the number of guard bands [29]. Mingwu Yao et al. presented a Mixed Initial Population Genetic Algorithm (MGA) to address the scheduling problem [35].

## 3. Routing Based on Flow Combinability

In this section, we present a routing method that is grounded in the concept of flow combinability. Initially, flows are grouped based on the greatest common divisor of their respective periods. Subsequently, a genetic algorithm is employed to facilitate routing according to flow combinability.

### 3.1. Flow Grouping Method Utilizing the Greatest Common Divisor

Kai Huang et al. demonstrated that if the greatest common divisor (GCD) of the periods of two flows is 1, it indicates a potential conflict when both flows traverse the same link, meaning these two flows cannot be combined [10]. In this paper, we categorize flows according to their GCDs, ensuring that uncombinable flows are separated into different groups. Furthermore, within any given group, the GCD of any two flows must be greater than 1.

Let F={1,2,…,m} be a set of flows. The flow grouping method based on the greatest common divisor is presented in Algorithm 1. In the first line, flows are sorted in ascending order by their periods, and the result is recorded as F_sort. Lines 4 to 8 identify the flow i with the smallest period that has not yet been grouped within F_sort. Lines 10 to 14 group the flows whose periods share a GCD greater than 1.
**Algorithm 1.** Flow Grouping Based on Greatest Common Divisor**Input:** flow set F={1,2,…,m}
**Output:** flow grouping classflow 1 Sort the flows in descending order based on their periods, denoted as $F\text{\_}sort=\{i_{1},i_{2},\ldots ,i_{m}\}$ 2 classflow=∅ 3 **while** there exist ungrouped flows within F_sort**do** 4        **for** $i=i_{1}\to i_{m}$ **do** 5             **if** i has not been grouped **then** 6                  **break** **7             end if** 8          **end for** 9          class_i={i} 10        **for** $k=i_{1}\to i_{m}$ **do** 11             **if** k has not been grouped and $gcd(T_{i},T_{k})\neq 1$ then 12                  class_i=class_i ∪{k} 13             **end if** 14         **end for** 15         classflow=classflow∪{class_i} 16 **end while** 17 **return** classflow


### 3.2. Route Selection Based on Genetic Algorithm

In this section, we present a routing method that utilizes a genetic algorithm [35]. This method is grounded in the definition of individual encoding, the construction of a fitness function for individuals, and the implementation of genetic operations. The tailored genetic algorithm is specifically designed to optimize the routing for each group of flows.

#### 3.2.1. Individual Coding

**Figure 1:**
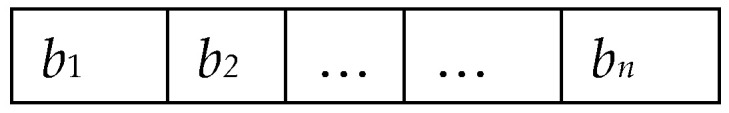
Routing selection encoding.

Let class_k denote the k-th group of classflow, which encompasses flows $f_{1}^{k}$, $f_{2}^{k}$, …, $f_{n}^{k}$. Each flow is associated with a corresponding set of feasible paths $PS_{1}$, $PS_{2}$, …, $PS_{n}$. The individual encoding employs a decimal representation in which each gene bit corresponds to a selected feasible path for each flow. An example of the individual coding is illustrated in Figure 1. The variable $n_{j}$ represents the number of the path selected by flow $f_{j}^{k}$, where 1≤j≤n.

#### 3.2.2. Fitness Function

The individual fitness function serves as the foundation for evaluating the quality of an individual within a genetic algorithm. The calculation of the individual fitness value should consider four critical factors: (1) flow combinability, (2) link load, (3) path length, and (4) the number of remaining feasible paths.

Let x represent an individual in the population, and let the components $x_{1},x_{2},\ldots ,x_{n}$ denote the number of paths chosen by these n flows. The corresponding paths are represented as $p_{1}^{x},p_{2}^{x},\ldots ,p_{n}^{x}$, respectively. Following the methodologies established in the literature [10], we define the sum of weights of all flows passing through link (u,v) within the path selected by the k-th group of flows as $SOW_{\left(u,v\right)}$. The formula for this calculation is presented in Equation (1).

(1) $${SOW}_{\left(u,v\right)}=\sum_{i\epsilon F_{\left(u,v\right)}^{x}}\frac{S_{i}}{T_{i}-\frac{T_{i}}{\mathrm{GCD}\left(u,v\right)}}$$

where $F_{\left(u,v\right)}^{x}=\left\{f_{i}^{k}|\left(u,v\right)\right\}$ is the link on $p_{i}^{x}$, 1≤i≤n denotes the set of flow, $T_{i}$ denotes the periods of *i*-th flow, and GCD(u,v) denotes the greatest common divisor of the periods of flows that traverse the link (u,v).

When ${SOW}_{\left(u,v\right)}$ is larger, it indicates that the bandwidth of the link (u,v) is occupied by a greater number of flows. Consequently, the flow combinability on this link decreases, resulting in lower schedulability for these flows traversing the same link. Conversely, when ${SOW}_{\left(u,v\right)}$ is smaller, fewer flows occupy the bandwidth of the link (u,v), thereby enhancing flow combinability for flows traversing the same link. This improvement facilitates higher schedulability for these flows as they pass through the same link.

However, if each flow exclusively prioritizes paths with high combinability during path selection, three potential issues may arise: (1) the number of network nodes that the time-triggered flow traversed might increase accordingly, thereby imposing a heavier load on the network; (2) this strategy could result in an excessive concentration of time-triggered flows passing through a single link; and (3) it might substantially reduce the number of remaining feasible paths for certain flows or even leave some flows without any viable paths. All these scenarios negatively affect the schedulability of the entire network. To address these challenges, the fitness function should consider ${SOW}_{\left(u,v\right)}$, the total path lengths, the number of flows traversing link (u,v), and the number of remaining feasible paths, simultaneously.

The total path lengths $L_{x}$ is calculated by Equation (2).

(2) $$L_{x}=\sum_{i=1}^{n}\sum_{(u,v)\in E}I_{u,v}^{p_{i}}$$

where $I_{u,v}^{p_{i}}$ is defined for 0 and 1 variables. If (u,v) is the link on $p_{i}^{x}$, $I_{u,v}^{p_{i}}$ = 1. Otherwise, $I_{u,v}^{p_{i}}$ = 0.

**Definition** **1.** *The intersection of paths* p *and q* *is defined as follows: If there exists a link* (u,v) *that is both a link on path* p *and q, then p∩q≠∅, otherwise p∩q=∅*.

Assume that all flows are categorized into h groups based on Algorithm 1. Let $r_{k}^{x}$ denote the path chosen by each flow in group k, while $r_{1,k-1}$ represents the paths selected by all flows from group 1 to group k−1. We define $N_{x}$ as the total number of remaining feasible paths for all flows from group k+1 to group h, which is contingent upon individual variable x. $N_{x}$ is calculated according to Equation (3).

(3) $$N_{x}=\sum_{i=k+1}^{h}\sum_{f\in classflow(i)}\sum_{p\in flowPath(f)}K_{f,p}\left(r_{k}^{x},r_{1,k-1}\right)$$

where classflow(i) represents the i-th flow group of classflow, and flowPath(f) consists of all simple paths associated with the flow f. $K_{f,p}\left(r_{k}^{x},r_{1,k-1}\right)$ is utilized to evaluate whether the simple path p is a feasible route for these flows. For a given simple path p of flow f, if there exists a flow $f^{*}$ in the first k groups with an associated path $p^{*}$, such that flows f and $f^{*}$ cannot be combined, and the intersection of paths p and $p^{*}$ is non-empty, then the simple path p is not a feasible path for flow f. $K_{f,p}\left(r_{k}^{x},r_{1,k-1}\right)$ can be determined using Equation (4).

(4) $$K_{f,p}\left(r_{k}^{x},r_{1,k-1}\right)=\left\{\begin{array}{cc} 0 & \exists \left(f^{*},p^{*}\right)\in r_{k}^{x}\cup r_{1,k-1}satisfy\gcd\left(T_{f}{,T}_{f^{*}}\right)=1\;and\;p\cap p^{*}\neq \emptyset \\ 1 & else \end{array}\right.$$

Let f(x) denote the fitness function of individual x. The definition of f(x) is as follows:

(5) $$f\left(x\right)=\sum_{(u,v)\in E}{SOW}_{u,v}+\alpha \times \sum_{(u,v)\in E}\left|{F_{u,v}}^{x}\right|+\beta \times N_{x}+L_{x}$$

The function f(x) is defined by four distinct formulas. The first formula quantifies the flow combinability, while the second reflects the magnitude of link load. The third formula indicates the number of remaining feasible paths, and the fourth assesses the length of the selected route path. Here, α represents a penalty factor associated with the total flow traversing through link (u,v); whereas β denotes a penalty factor related to the count of available remaining paths.

#### 3.2.3. Feasible Path for Flow

Since two uncombinable flows traversing the same link will inevitably collide, effective scheduling in time-sensitive networks cannot be guaranteed. As a result, not all simple paths for a flow within the network are feasible. For instance, if the route selected by flow j includes link (u,v), and flow i is incompatible with flow j, then any path for flow i that contains link (u,v) becomes invalid. Consequently, it is essential to determine which paths are feasible for each specific flow. This section presents the methodology for identifying such feasible paths.

Let us first define a function named Optional Path Set Construction, which is designed to identify a simple path that does not contains specified link. The detailed implementation of this function is presented in Algorithm 2. The function determines whether each simple path in flow i includes any link from the set of ablist (line 2~12). If a simple path contains a link from the ablist, this path is marked with 0. Otherwise, it is marked with 1. Subsequently, the marker of each simple path of flow i is analyzed, and the path designated with the value of 1 is considered an optional path for flow i (line 13~16). If a simple path contains a link from the ablist, this path is marked with 0. Otherwise, it is marked with 1. Subsequently, the marker of each simple path of flow i is analyzed, and the path designated with the value of 1 is considered an optional path for flow i (line 13~16).
**Algorithm 2** Optional Path Set Construction**Input:** A simple path set of flowPath, flow i, a link set of ablist **Output:** Optional path for flow i 1 feaspath=∅, feaspathflag=∅ 2 **for** k=1→len(flowPath[i]) **do** 3       flag=1 4      **for** j=1→len(ablist) **do** 5            a=ablist[j][0] 6            b=ablist[j][1] 7            **if** (a,b) *in* flowPath[i][k] **then** 8                  flag=0 9            **end if** 10      **end for** 11      feaspathflag=feaspathflag∪{flag} 12 **end**
**for** 13 **for** k=1→len(feaspathflag) **do** 14      **if** feaspathflag[k]=1 **then** 15             feaspath=feaspath∪{flowPath[i][k]} 16      **end if** 17 **end for** 18 **return** feaspath


Based on Algorithm 2, we propose a function referred to as Feasible Path Set Construction, as shown in Algorithm 3, which is specifically designed to identify the feasible paths for each flow within the k-th group of classflow. This function consists of five parameters: the simple path for all flows (flowPath), the flows set (F), the flows grouping (classflow), the order number of groups (k), and the selected paths of all flows in the preceding k−1 group (haveselect). When k=1, there are no selected paths for flow, that is, haveselect=∅. Consequently, the feasible paths for each flow within this group comprise all its simple paths in the network. When k>1, lines 9 to 25 of the function are employed to identify the feasible path for each flow within the group. Firstly, we obtain a flow from group k, denoted as *a* (line 12). Additionally, we acquire a flow from the set of haveselect, represented as b (line 13). If a and b cannot be combined, each link along the path of flow b is added to the set of ablist (line 14~21). Subsequently, Function 1 is invoked to identify a viable path for flow *a* (line 23).
**Algorithm 3** Feasible Path Set Construction**Input:** flowPath*,* F,classflow,k,haveselect **Output:** Feasible path for each flow within the k-th group 1 feaspathlist=∅ 2 **if** *k* = 1 **then** 3          **for** i=1→len(classflow[k]) **do** 4                j=classflow[k][i] 5                feaspathlist=feaspathlist∪flowPath[j] 6          **end for** 7          **return** feaspathlist 8 **end if** 9 **for** i=2→len(classflow[k]) **do** 10     ablist=∅ 11     **for** j=1→len(haveselect) **do** 12          a=classflow[k][i]
13          b=haveselect[j][0]
14          **if** $gcd(T_{a},\;T_{b})=1$ **then** 15                 path=haveselect[j][1] 16                 **for** x=1→len(path) **do** 17                        **if** [path[x], path[x+1]] not in ablist **then** 18                               ablist=ablist∪[path[x], path[x+1]] 19                        **end if** 20                 **end for** **21          end if** **22     end for** **23**     templist=feasible_path(flowPath, classflow[k][i], ablist) 24     feaspathlist=feaspathlist ∪templist 25 **end for** 26 **return** feaspathlist


#### 3.2.4. Route Selection Algorithm

We present a route selection method for each flow using a genetic algorithm, as detailed in Algorithm 4. This algorithm employs a set named haveselect to record the paths chosen by each flow, with this set being initially as empty. The *k*-th iteration of the algorithm’s outer loop focuses on identifying the route for the flow within the group k. Each iteration starts by invoking the Feasible Path Set Construction function to determine the feasible paths for each flow within group k (line 3). Subsequently, the parameters of the genetic algorithm are initialized, and the initial population is generated (line 4~5). The inner loop executes genetic operations such as selection, crossover, and mutation, while also updating the best individual (line 6~9). Once the paths for group k have been determined, the haveselect set is updated accordingly (line 10).
**Algorithm 4** Route selection based on genetic algorithm**Input:** the simple path for all flows (flowPath), flows set (F), flows grouping (classflow) **Output:** Route for each flow 1 haveselect=∅ 2 **for** k=1→len(classflow) **do** 3         feaspathlist=compute_feaspathlist(flowPath, F, clnassflow, k, haveselect) 4         Initialize population size, crossover probability, mutation probability 5         Initialize the population and calculate the optimal individual 6         **for** i=1→maxiter **do** 7              Implement selection, crossover and mutation operations 8              Update the optimal individual 9         **end for** 10       Update haveselect based on the optimal individual 11 **end for** 12 **return** haveselect


## 4. Scheduling for TSN Based on Differential Evolution Algorithm

### 4.1. Scheduling Problem and Modeling

In the preceding section, we have introduced a routing selection method that considers flow combinability. Once the routing for all flows has been established, it becomes crucial to determine the transmission timing of each frame within every flow at each node along its path, ensuring that all frames of each flow can be transmitted and received within the designated time during the super period, while also guaranteeing that no two transmissions on a link (in the same direction) overlap. This concept is referred to as network scheduling.

In this section, we propose a novel approach to find a feasible scheduling scheme for TSN based on the differential evolution algorithm. We assume that the time sensitive network satisfies zero jitter and no frame loss. Let the path of flow i be denoted as $p_{i}=n_{1}^{i}\to n_{2}^{i}\to ,\cdots ,{\to n}_{l_{i}}^{i}$. The duration required to transmit flow i from any given node to its adjacent nodes is represented as $W_{i}$. The latest deadline by which the listener must acquire the data is denoted as $D_{i}$. The period of flow i is indicated by $T_{i}$ and the hyper-period of the network is represented as HP. Let $S_{k,t}^{i}$ denote the sending time of the *t*-th frame for flow i at the *k*-th node. A feasible scheduling solution of TSN must satisfy the following four constraints:

(1) Precedence Constraint

A frame transmission along the path must satisfy the precedence constraint, meaning that the transmission time of a frame at node k+1 cannot be earlier than the sum of the transmission time of the frame at node k and the propagation delay between two adjacent nodes. This constraint can be described by Formula (6).

(6) $$S_{k+1,t}^{i}\geq S_{k,t}^{i}+W_{i}$$

where $1\leq k\leq l_{i}-1$, $l_{i}$ represents the total number of nodes traversed by flow i along its path.

(2) Deadline Constraint

The time interval between the listener and the talker node must not exceed $D_{i}$. This constraint can be described by Formula (7).

(7) $$S_{l_{i}-1,t}^{i}+W_{i}-S_{1,t}^{i}\leq D_{i}$$

(3) Period Constraint

The reception time of the final frame in flow i must not surpass the hyper-period. This constraint can be described by Formula (8).

(8) $$S_{l_{i}-1,\frac{HP}{T_{i}}}^{i}+W_{i}\leq HP$$

(4) Resource Constraint

For each pair of different frames on a link, one frame must be transmitted only after the preceding frame has been fully transferred. For any two flow $i_{1}$ and $i_{2}$, if both flows traverse the link (u,v), any $\alpha \in \{0,1,\cdots ,\frac{lcm\left(T_{i_{1}},T_{i_{2}}\right)}{T_{i_{1}}}\}$ and any $\beta \in \{0,1,\cdots ,\frac{lcm\left(T_{i_{1}},T_{i_{2}}\right)}{T_{i_{2}}}\}$ must satisfy the following constraint:

(9) $$S_{u,1}^{i_{1}}+\alpha \cdot T_{i_{1}}\geq S_{u,1}^{i_{2}}+\beta \cdot T_{i_{2}}+W_{i_{2}}\vee \;S_{u,1}^{i_{2}}+\beta \cdot T_{i_{2}}\geq S_{u,1}^{i_{1}}+\alpha \cdot T_{i_{1}}+W_{i_{1}}$$

where $i_{1}<i_{2}$.

**Theorem** **1.** *If the transmission time of the first frame of flow i satisfies the precedence constraint, it follows that the transmission time of the t-th frame of flow i also satisfies the precedence constraint, where $2\leq t\leq \frac{HP}{T_{i}}$*.

The proof is given in Appendix A.1.

**Theorem** **2.** *If the first frame of flow *i *satisfies the deadline constraint, the t-th frame of flow i* *also satisfies the deadline constraint, where* $2\leq t\leq \frac{HP}{T_{i}}$.

The proof is given in Appendix A.2.

**Theorem** **3.** *If $S_{1,1}^{i}+\left(\frac{HP}{T_{i}}-1\right)\cdot T_{i}+(l_{i}-1)\cdot W_{i}\leq HP$* *, flow i meets the period constraint.*

The proof is given in Appendix A.3.

**Theorem** **4.** *Let the paths of flows* $i_{1}$ *and $i_{2}$ share a common link (u, v), where the serial number of node* u *on the path of flow* $i_{1}$ *is denoted as $k_{1}$,* *and the serial number of node* v *on the path of flow* $i_{2}$ *is denoted as $k_{2}$. The expression of Formula (9) can be equivalently represented as Formula (10), which is detailed as follows:*

(10) $$\begin{array}{cc} S_{1,1}^{i_{1}}+\left(k_{1}-1\right)\cdot & W_{i_{1}}+\alpha \cdot T_{i_{1}}\\ & \geq S_{1,1}^{i_{2}}+\left(k_{2}-1\right)\cdot W_{i_{2}}+\beta \cdot T_{i_{2}}+W_{i_{2}}\vee S_{1,1}^{i_{2}}+\left(k_{2}-1\right)\cdot W_{i_{2}}\\ & +\beta \cdot T_{i_{2}}\geq S_{1,1}^{i_{1}}+\left(k_{1}-1\right)\cdot W_{i_{1}}+\alpha \cdot T_{i_{1}}+W_{i_{1}} \end{array}$$

The proof is given in Appendix A.4.

According to the conclusions drawn from the aforementioned four theorems, it is sufficient to determine the transmission time of the first frame of each flow at the talker. Subsequently, we can compute the transmission times for this frame at other nodes and ascertain the transmission times for additional frames at each node. This process enables us to evaluate whether scheduling is feasible. Consequently, our scheduling algorithm requires only solving for the transmission time of the first frame at the talker. Thus, this approach significantly reduces the complexity associated with addressing this problem.

**Theorem** **5.** *Let $n_{1},n_{2},\cdots ,n_{m}$* *represent the number of nodes in the path of flow* $i_{1},i_{2},\cdots ,i_{m},$ *respectively. Assume that for the links (*$u_{1}$*,$v_{1}$), ($u_{2}$,$v_{2}$), …, ($u_{h}$,$v_{h}$), each link carries two or more flows, denoted respectively as {$i_{1}^{1}$, $i_{2}^{1}$, …$,i_{k_{1}}^{1}$* *},{$i_{1}^{2}$, $i_{2}^{2}$, …$,i_{k_{2}}^{2}$* *}, …,{$i_{1}^{h}$, $i_{2}^{h}$, …$,i_{k_{h}}^{h}$* *}. Let the number of constraints that a feasible scheduling must satisfy be denoted as CN. Consequently, it follows that the value of CN is equivalent to the value presented in Formula (11).*

(11) $$\sum_{i=1}^{m}\left(n_{i}-2\right)+2m+\sum_{j=1}^{h}\sum_{x=1}^{k_{j}}\sum_{y=1\wedge x\neq y}^{k_{j}}\frac{{\left[lcm(T_{i_{x}^{j}},T_{i_{y}^{j}})\right]}^{2}}{T_{i_{x}^{j}}\cdot T_{i_{y}^{j}}}$$

The proof is given in Appendix A.5.

### 4.2. Gene Coding

**Figure 2:**
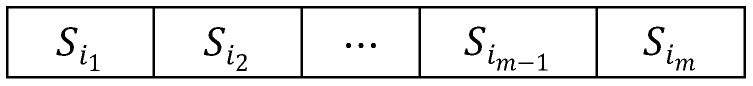
Gene coding for scheduling. $S_{i_{1}}$, $S_{i_{2}}$, ⋯, $S_{i_{m-1}}$, $S_{i_{m}}$ represent the transmission time of flows $i_{1},i_{2},\cdots ,i_{m}$ at the talker.

According to Theorems 1 to 4, once the transmission time of the first frame in a flow at the sending node is established, it becomes possible to determine the transmission times for all frames within that flow across each node. Subsequently, this allows us to assess whether the scheduling of the flow adheres to relevant constraints. Therefore, the problem of scheduling in time-sensitive networks fundamentally revolves around determining the transmission time of the first frame for each flow at its respective talker. To address this issue, we can represent the time-sensitive network scheduling encoding for flows $i_{1},i_{2},\ldots ,i_{m}$ as a real number encoding with a length of *m*, as shown in Figure 2.

According to the genetic code, the solution to scheduling problem fundamentally involves determining the transmission time of the first frame for each flow at the talker. A larger range of transmission time results in an extended algorithmic solution time, whereas a smaller range leads to a reduced solution time. We present the following theorem concerning the upper limit of this value.

**Theorem** **6.** *Given that the path of flow* $i_{k}$ *is denoted as $p_{i_{k}}$* *= $n_{1}^{i_{k}}\to n_{2}^{i_{k}}\to ,\cdots ,{\to n}_{l_{i_{k}}}^{i_{k}}$, the duration required to transmit flow* $i_{k}$ *from any given node to its adjacent nodes is represented as $W_{i_{k}}$, and the deadline of flow* $i_{k}$ *is denoted as $D_{i_{k}}$, it can be concluded that value of* $S_{i_{k}}$ *must not surpass* $D_{i_{k}}-W_{i_{k}}\cdot (l_{i_{k}}-1)$ *, where $1\leq k\leq i_{m}$*.

The proof of is given in Appendix A.6.

To ascertain whether the flow scheduling adheres to the relevant constraints, it is essential to determine the transmission time of each flow at every node. Let us denote the transmission time of the first frame of flow i at the talker $n_{1}^{i}$ as $S_{i}$. Consequently, the transmission times for this frame at nodes $n_{2}^{i},\cdots ,n_{l_{i}-1}^{i}$ can be expressed as $S_{i}$, $S_{i}+W_{i}$, …, and $S_{i}+(l_{i}-2)*W_{i}$, respectively. Therefore, based on the encoding $S_{i_{1}}$, $S_{i_{2}}$, ⋯*,* $S_{i_{m-1}}$, $S_{i_{m}}$, each flow can be derived at each node during transmission time.

Let $X=[S_{i_{1}},S_{i_{2}},\cdots ,S_{i_{m}}]$ represent the sending times of the first frame of each flow at the talker. The transmission times for each flow at every node are determined using Algorithm 5. Let Y be utilized to store the transmission times of each flow across all nodes. A for loop is employed to traverse through each flow, where X[k] denotes the transmission times of flow $i_{k}$ at the talker. The while loop is designed to compute the transmission times for the subsequent nodes of flow $i_{k}$ (line 5~8). Here, $W_{i_{k}}$ signifies the transmission time of flow $i_{k}$ in an adjacent node.
**Algorithm 5** Computing the transmission time of each frame at every node along its path**Input:** The transmission time of the first frame X **Output:** The transmission time of each frame at every node along its path Y1 Y=[ ] 2 **for** k=1→m **do** 3         Y.append(X[k]) 4         j=1 5         **while** $j<=len(p_{i_{k}})-1$ **do** 6              *Y*.append($Y[len(Y)]+W_{i_{k}}$) 7              j=j+1 8         **end while** 9 **end for** 10 **return** Y


### 4.3. The Fitness Function and Its Calculation

According to Theorem 5, we gain insight into the number of constraints that a feasible scheduling solution must fulfill. Consequently, we regard the quantity of satisfiable constraints as the fitness function for the differential evolution algorithm. The fitness function is defined as Equation (12).

(12) $$f\left(X\right)=\phi \left(X\right)+\omega \left(X\right)+\eta \left(X\right)+\sigma (X)$$

where ϕ(X) represents the number of constraints that individual X satisfies the precedence constraint, ω(X) denotes the number of constraints that X meets the deadline constraint, η(X) indicates the number of constraints that X satisfies period constraint, and σ(X) signifies the number of constraints that X adheres to with respect to the resource constraint.

If a link is traversed by multiple flows, it is referred to as a shared link. The key of determining the value of f(X) lies in calculating the quantity of constraints that X meets the resource constraint. In order to compute the number of constraints that X satisfies the resource constraint, we must identify all shared links. Firstly, an algorithm is developed to address the link information according to the path of all flows, as detailed in Algorithm 6. The link information is represented by a three-tuple: $\left[i_{r},(p_{r,j},p_{r,j+1}),num\right]$, where $i_{r}$ denotes the flow number, $\left(p_{r,j},p_{r,j+1}\right)$ represents the *j*-th link on path $p_{r}$, and num denotes the number associated with this link. Each iteration of the outer loop begins by calculating the length of the path (line 3). Subsequently, it obtains every link of the path and assigns its numerical identifiers (line 4~7).
**Algorithm 6** Deriving Link Information**Input:** $\mathrm{Flow}\;i_{1},\;i_{2},\ldots ,i_{m}$ and its path $p_{1},\;p_{2},\ldots ,p_{m}$ **Output:** Link information path_infor 1 path_infor=φ, num=1 2 **for** r=1→m **do** 3         $k=len(p_{r})$ 4         **for** j=1→k **do** 5              $path\text{\_}infor=path\text{\_}infor\;\cup \{[i_{r},\;(p_{r,j},\;p_{r,j+1}),\;num]\}$ 6              num=num+1 7         **end for** 8 **end for** 9 **return** path_infor


Given flow $i_{1},i_{2},\ldots ,i_{m}$ and its path $p_{1},p_{2},\ldots ,p_{m}$, we present a way to obtain shared links based on the link information, which is determined by Algorithm 6. Algorithm 7 provides a detailed description of the process for identifying shared links. In each iterate, creating a temporary set named temp and this set contains the iterated tuple (line 2). It then retrieves the flow and the link associated with this tuple (line 3~4). Subsequently, it examines the flow and the link present in other tuples. If two different flows share a common link, such a tuple is added to temp (line 6~12). In cases where multiple elements exist within temp, it is incorporated into the set of conflict_infor (line 14~16).
**Algorithm 7** Finding Shared Link**Input:** Link information path_infor **Output:** The set of shared link conflict_infor 1 **for** i=1→len(path_infor) **do** 2        temp={path_infor[i]} 3        f1=path_infor[i][0] 4        l1=path_infor[i][1] 5        j=i+1 6        **while** j<=1→len(path_infor) **do** 7                f2=path_infor[j][0] 8                l2=path_infor[j][1] 9                **if** $f_{1}\neq f_{2}$ and $l_{1}=l_{2}$ **then** 10                     temp=temp∪{path_infor[j]} 11              **end if** 12              *j* = *j* + 1 13       **end while** 14       **if** len(temp)>1 **then** 15              conflict_infor=conflict_infor∪temp 16       **end if** 17 **end for** 18 **return** conflict_infor


Based on the shared link derived from Algorithm 7, we present the algorithm for calculating the value of f(X), as illustrated in Algorithm 8. Firstly, Algorithm 5 is applied to compute the transmission time of each frame at every node along its path (line 2). Then, the corresponding subscript in Y for the transmission time of flow $i_{k}$ at its talker is determined (line 4). Subsequently, the quantity of the precedence constraint that a flow must satisfy is calculated (lines 6–9), the quantity of the deadline constraint that a flow must meet is established (line 10), and the quantity of the period constraint that a flow must adhere to is evaluated (lines 11–13). For each set within the conflict_infor, consider any two tuples contained in that set. First, extract the flow associated with these two tuples. Subsequently, obtain the corresponding subscript in Y for the transmission time of both flows at the talker (line 19~20). Finally, whether flows $i_{x}$ and $i_{y}$ satisfy the resource constraint is assessed and the quantity of this constraint is computed (line 21~27).
**Algorithm 8** Computing the fitness of individual**Input:** Individual X **Output:** The fitness of individual 1 sum=0 2 Computing the transmission time of each frame by Algorithm 5 and store it in Y 3 **for** k=1→m **do** 4       **if** k=1 **then** j=1 **else** $j=j+len(p_{i_{k}})-1$ **end if** 5       t=j, s=j 6       **while** $t<=j+len(p_{i_{k}})-1$ **do** 7              **if** $Y[t]>=Y[t-1]+W_{i_{k}}\;$ **then** sum=sum+1 **end if** 8              t=t+1 9       **end while** 10     **if** $Y[t-1]+W_{i_{k}}-Y[s]<=W_{i_{k}}$ **then** sum=sum+1 **end if** 11     **if** Ys+HPTik−1*Tik+lenpik∗Wik≤HP **then** 12            sum=sum+1 13     **end if** 14 **end for** 15 **for** each **in** conflict_infor **do** 16   **for** tuple1 **in** each **do**
17     **for** tuple2 **in** each **do** 18            **if** tuple1≠tuple2
**then** 19              $i_{x}=tuple1[1],\;i_{y}=tuple2[1]$ 20              $x_{1}=tuple1[3],\;x_{2}=tuple2[3]$ 21              **for** $k1=0\to lcm(T_{i_{x}},\;T_{i_{y}})/T_{i_{x}}-1$ **do** 22                **for** $k2=0\to lcm(T_{i_{x}},\;T_{i_{y}})/T_{i_{y}}-1$ **do** 23                  **if** $Y[x1]+k1*T_{i_{x}}>=Y[x2]+k2*T_{i_{y}}+W_{i_{y}}\;or\;Y[x2]+k2*T_{i_{y}}>=Y[x1]+k1*T_{i_{x}}+W_{i_{x}}$ **then** 24                    sum=sum+1 25                  **end if** 26                **end for** 27              **end for** 28            **end if** 29     **end for** 30   **end for** 31 **end for** 32 **return** sum


### 4.4. Optimization Objective of Differential Evolution Algorithm

Based on the preceding analysis, it can be concluded that if the value of f(X) equals to the number of constraints CN that a feasible scheduling solution must satisfy, then the individual X is indeed a feasible scheduling solution. Therefore, we utilize a differential evolution algorithm to maximize the value of f(X). During the iterative process of this algorithm, the execution will terminate under one of two conditions: either f(X) equals CN, or the maximum iteration limit is reached. Upon completion of the algorithm, if f(X) equals CN, we provide the scheduling results for each flow based on X. Conversely, if f(X)<CN, it indicates that the algorithm cannot find a feasible scheduling for TSN.

### 4.5. Differential Evolution Algorithm for TSN Scheduling

The differential evolution algorithm is an efficient global optimization algorithm [36]. Additionally, it is a population-based heuristic search algorithm, where each individual within the population represents a solution vector. The evolutionary process of differential evolution closely resembles that of genetic algorithms, involving mutation, crossover, and selection operations. However, the specific definitions and implementations of these operations differ significantly from those in genetic algorithms.

#### 4.5.1. Scheduling Coding

Given that the scheduling coding of flows $i_{1},i_{2},\cdots ,i_{m}$ is represented as [$S_{i_{1}}$,$S_{i_{2}}$, ⋯, $S_{i_{m}}$], according to Theorem 6, the value range of $S_{i_{k}}$ extends from 0 to $D_{i_{k}}-W_{i_{k}}\cdot (l_{i_{k}}-1)$, where 1≤k≤m. The initial values of the component $S_{i_{k}}$ is computed using the following formula:

(13) $$S_{i_{k}}=randint(0,D_{i_{k}}-W_{i_{k}}\cdot (l_{i_{k}}-1))$$

Here, randint(a,b) denotes a random integer between a and b.

#### 4.5.2. Operators of Differential Evolution Algorithm

The operations of differential evolution algorithms include mutation, crossover, and selection operations.

(1) Mutation

For each individual *x_i_* within the population, its mutation vector is generated based on the following formula:

(14) $$v_{i}=x_{r1}+F\cdot (x_{r2}-x_{r3})$$

where $x_{r1}$, $x_{r2}$, $x_{r3}$ were randomly selected from the population and i≠r1≠r2≠r3. The parameter F is utilized to regulate the extent of bias amplification. In this paper, we employ an adaptive adjustment strategy for the F value as follows:

(15) $$\gamma =1-\frac{maxiter}{maxiter+1-k}$$

(16) $$F=F_{0}\cdot 2^{\gamma }$$

where maxiter is the maximum number of iterations of DE algorithm, k is the number of iterations, and $F_{0}\in \left[0.2,0.5\right]$. $F_{0}$ usually takes 0.4.

(2) Crossover

The objective of crossover is to recombine the components of an individual $x_{i}$ and its mutation individual $v_{i}$ in order to generate a new individual. The crossover process is conducted as follows:

(17) $$u_{i,j}=\left\{\begin{array}{cc} v_{i,j} & \;r\leq CR\;⋁\;randint\left(1,D\right)=j\\ u_{i,j} & \;r>CR\;\wedge \;randint\left(1,D\right)\neq j \end{array}\right.$$

where *CR* is the crossover probability and *D* is the number of individual components.

(3) Selection

According to the greedy criterion, the differential evolution algorithm compares the new individual $u_{i}$ and the original individual $x_{i}$ obtained by mutation and crossover, and the one with the highest fitness value is selected as the individual of the next generation of the population. The selection is as follows:

(18) $$x_{i}^{t+1}=\left\{\begin{array}{cc} u_{i}^{t} & f\left(u_{i}^{t}\right)>f\left(x_{i}^{t}\right)\\ x_{i}^{t} & else \end{array}\right.$$

## 5. Simulation Experiment

In this section, we evaluate the validity of the proposed flow-combinability-based genetic algorithm for routing in TSN and the developed differential evolution algorithm for scheduling in TSN. Specifically, we compare the proposed algorithm with the shortest path-based ILP method [29] denoted as SP-ILP, the degree of conflict (DoC)-aware flows partitioning-based ILP method [32] denoted as DA-ILP, and the joint routing and scheduling (JRS)-based ILP method [37] denoted as JRS-ILP. The proposed algorithm was implemented using Python 3.8, and all simulations were executed on a PC equipped with an Intel^®^ Core (TM) i7-10510U CPU (1.80 GHz base frequency, boosting up to 2.30 GHz) 16 GB RAM, and running Windows 10.

### 5.1. TSN with Each Flow Has a Unique Shortest Path and All Flows Can Be Combined

In this series of experiments, we investigate whether each flow in TSN can transmit information via the shortest path and be successfully scheduled, assuming that the shortest path for each flow is unique and all flows are combinable.

#### 5.1.1. Experiment 1

**Figure 3:**
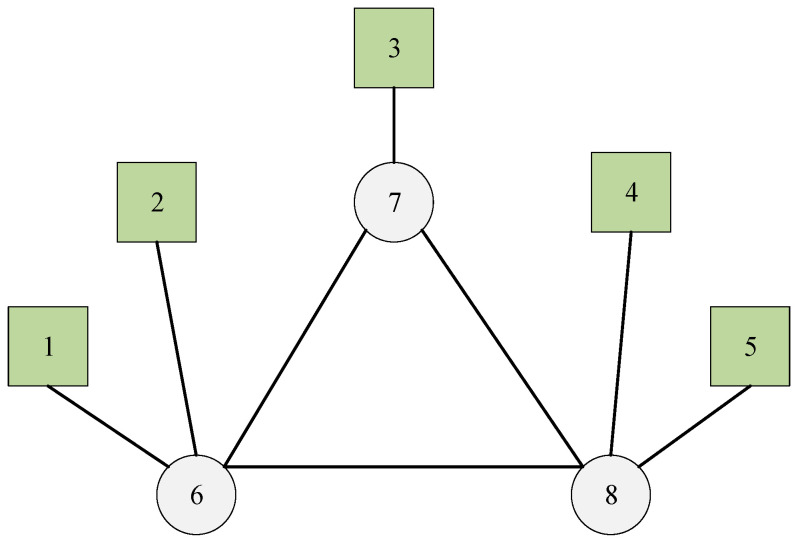
The network of Experiment 1. The circles represent the switches, the squares represent the terminals, and the numbers are the node sequence numbers.

In this case, we first examine a small-scale mesh network illustrated in Figure 3, which has three switches, five end stations, and three flows. The set of flows is defined as F={0,1,2}, with their corresponding parameters detailed in Table 1. The delay of the switch is 1 unit. For flow0, the transmission time between adjacent nodes is 35 units; conversely, both flow1 and flow2 have a transmission time of 24 units between adjacent nodes.

**Figure 4:**
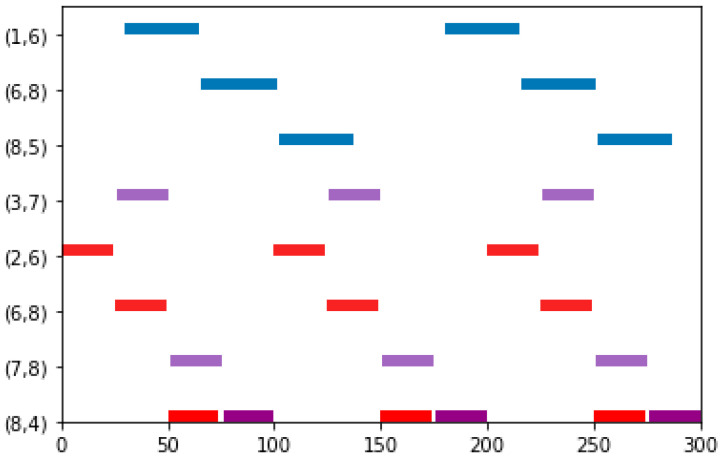
The schedule Gantt chart of Experiment 1. The schedult results of flow0, flow1, and flow2 are indicated by bule, red, and purple, respectively.

The proposed method effectively schedules each flow via the shortest path. The Gantt chart depicting the schedule is presented in Figure 4, where flow0, flow1, and flow2 are indicated by blue, red, and purple, respectively. In this experiment, all flows can be successfully scheduled using SP-ILP, DA-ILP, and JRS-ILP methods.

#### 5.1.2. Experiment 2

**Figure 5:**
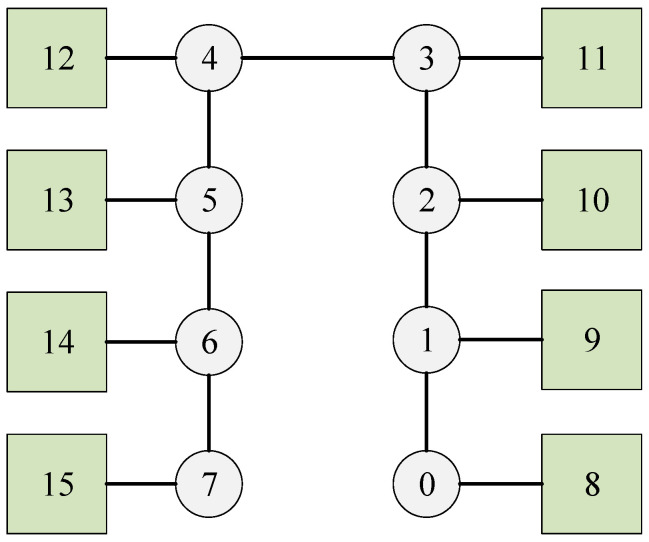
The network of Experiment 2. The circles represent the switches, the squares represent the terminals, and the numbers are the node sequence numbers.

In this case, we examine a line network illustrated in Figure 5, which has eight switches, eight end stations, and nine flows. The set of flows is defined as F={0,1,2,3,4,5,6,7,8,9}, with their corresponding parameters detailed in Table 2. Each switch incurs a delay of 1 unit, and each flow is transmitted between two adjacent nodes with a transmission time of 24 units.

**Figure 6:**
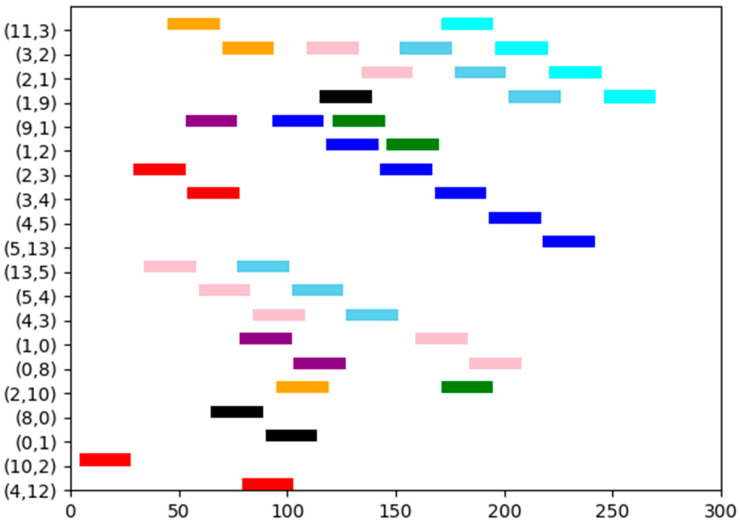
The schedule Gantt chart of Experiment 2. The schedule results of flows 0 to 8 are respectively indicated by cyan, blue, sky-blue, purple, pink, orange, black, brown, and red.

The proposed method effectively schedules each flow by utilizing the shortest path. As illustrated in Figure 6 through a Gantt chart, flows 0 to 8 are respectively indicated by cyan, blue, sky-blue, purple, pink, orange, black, brown, and red. In this scenario, each flow follows a distinct path. Moreover, due to the seamless integration of all flows, it is evident that each flow can also be scheduled using DE, SP-ILP, DA-ILP, and JRS-ILP methods.

#### 5.1.3. Experiment 3

**Figure 7:**
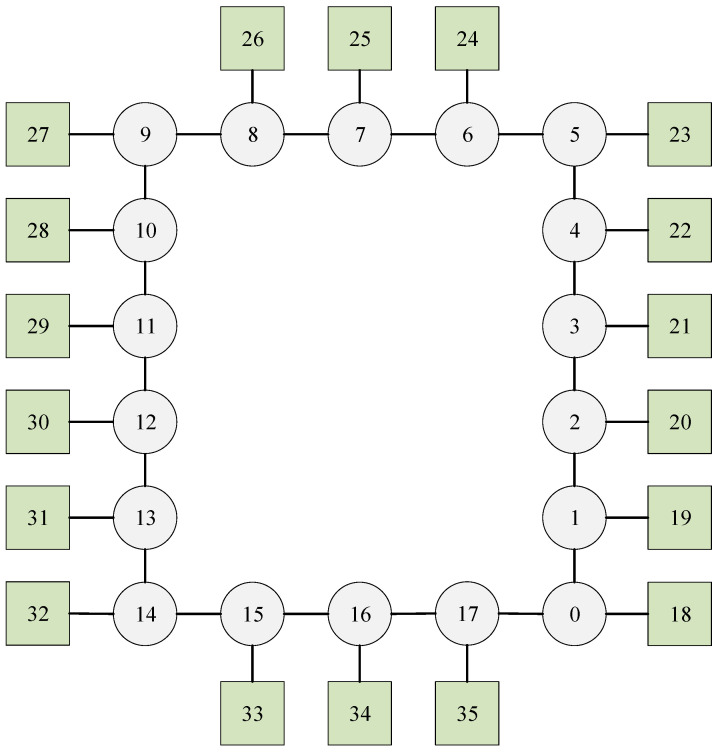
The network of Experiment 3. The circles represent the switches, the squares represent the terminals, and the numbers are the node sequence numbers.

In this case, we examine a ring network illustrated in Figure 7, which has 18 switches, 18 end stations, and 10 flows. The set of flows is defined as F={0,1,2,3,4,5,6,7,8,9}, with their corresponding parameters detailed in Table 3. Each switch incurs a delay of 1 unit, and each flow is transmitted between two adjacent nodes with a transmission time of 35 units.

**Figure 8:**
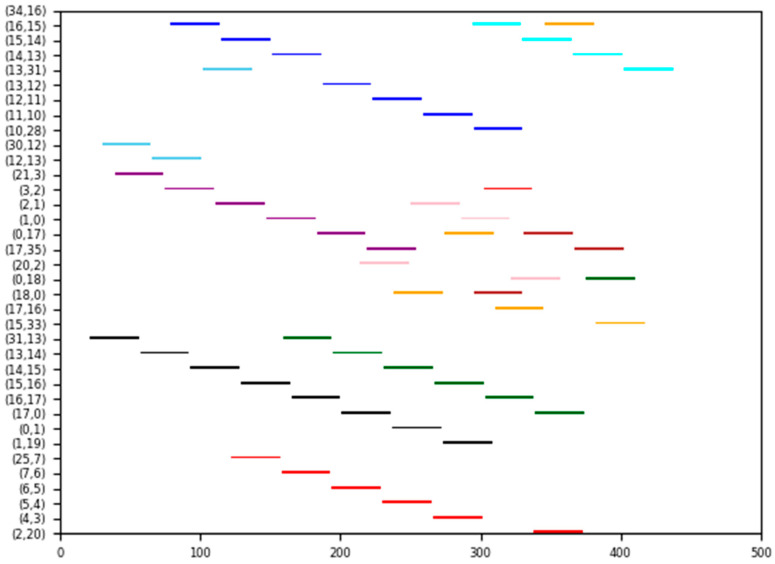
The schedule Gantt chart of Experiment 3. The schedule results of flows 0 to 9 are represented by cyan, blue, sky-blue, purple, pink, orange, black, brown, red, and green, respectively.

The method proposed in this paper ensures that each flow is scheduled along the shortest path with a probability of 100%. Specific routing details for each flow are provided in Table 3. Additionally, the Gantt chart depicting the schedule is shown in Figure 8, where flows 0 to 9 are represented by cyan, blue, sky-blue, purple, pink, orange, black, brown, red, and green, respectively.

In this case, all flows can be effectively combined, and each flow is successfully scheduled using the proposed method, as well as the SP-ILP method and the JRS-ILP method. However, it is important to note that the JRS-ILP method without optimization of path length does not guarantee the successful scheduling of each flow along the shortest path. The DA-ILP method could not find a feasible solution, as the DoC-aware routing method is not always stable.

### 5.2. TSN with Each Flow Has a Unique Shortest Path but Not All Flows Can Be Combined

In this section, we investigate the TSN scenario where each flow is assigned a unique shortest path, yet not all flows can be combined. Specifically, our aim is to quantify the number of flows that can successfully transmit information via their shortest path, assuming that each flow is scheduled without conflict.

**Figure 9:**
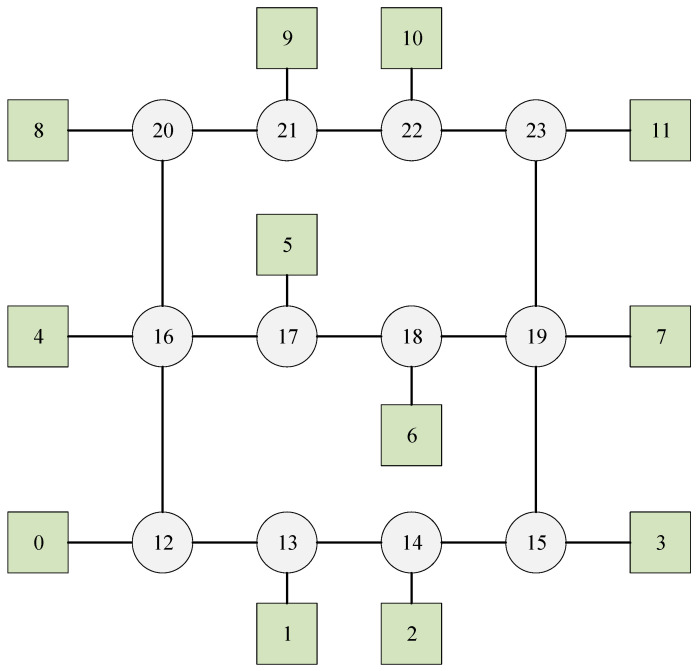
The network of Experiment 4. The circles represent the switches, the squares represent the terminals, and the numbers are the node sequence numbers.

In this experiment, we examine a mesh network illustrated in Figure 9, which has 12 switches, 12 end stations and 5 flows. The set of flows is defined as F={0,1,2,3,4}, with their corresponding parameters detailed in Table 4. Each switch incurs a delay of 0.1 units, and the transmission time between two adjacent nodes for each flow is 1 unit.

**Figure 10:**
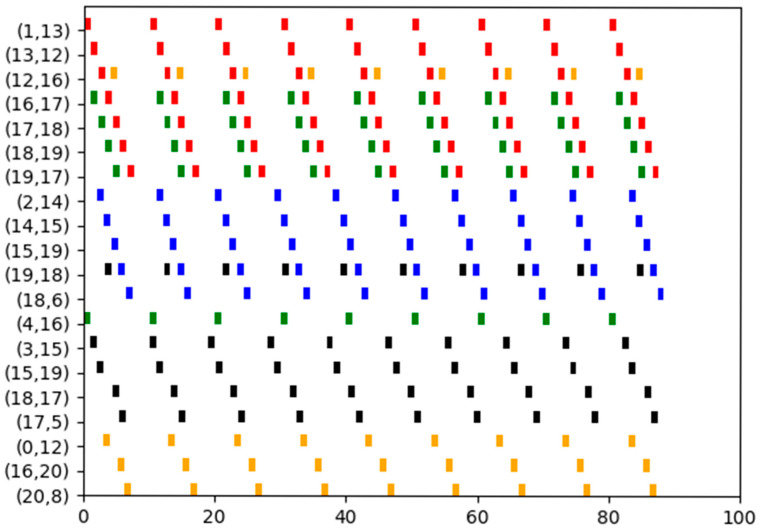
The schedule Gantt chart of Experiment 4. The schedule results of flow 0 to 4 are indicated in red, blue, green, black, and orange, respectively.

According to Algorithm 4, the path selected by flow0 corresponds to the second shortest path, specifically: 1-13-12-16-17-18-19-7. By contrast, all other flows adopt the shortest paths. Moreover, four flows transmit data via the shortest path. Based on these routing results, effective flow scheduling can be achieved by applying Algorithm 8. The Gantt chart depicting this schedule is shown in Figure 10, where flow0 is indicated in red, flow1 in blue, flow2 in green, flow3 in black, and flow4 in orange.

The shortest paths for both flow0 and flow1 include the links (14,15). However, it is not possible to combine flow0 and flow1. Consequently, the successful implementation of flow scheduling under the routing policy based solely on the shortest path cannot be achieved, leading to a probability of successful scheduling of zero.

In this instance, the SP-ILP method and DA-ILP method could not find a successful schedule. The reason is that, whether it is the SP-ILP method or the DA-ILP method, they both belong to the fixed route scheduling method, that is, the implementation adopts a specific method to set a fixed route for each flow before scheduling, without comprehensively considering the route and scheduling. However, regarding the JRS-ILP method without optimization of path length, while it is true that every flow can be scheduled successfully during each run, there is no assurance that the number of flows transmitting messages over the shortest path will simultaneously reach four.

### 5.3. TSN with Some Flows Have Multiple Shortest Paths and Not All Flows Can Be Combined

**Figure 11:**
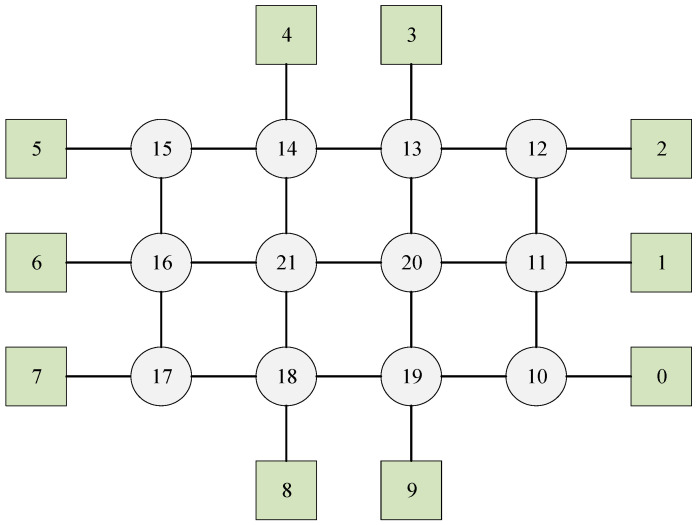
The network of Experiment 5. The circles represent the switches, the squares represent the terminals, and the numbers are the node sequence numbers.

In this section, we explore the TSN scenario in which certain flows have multiple shortest paths available, and not all flows can be simultaneously combined. We mainly evaluate the probability of successfully scheduling each flow along its shortest path. To illustrate this, we analyze a mesh network depicted in Figure 11, which has 12 switches, 10 end stations, and six flows. The set of flows is defined as F={0,1,2,3,4,5}, with their corresponding parameters detailed in Table 5. In this setup, each switch incurs a delay of 0.1 units, and the transmission time between two adjacent nodes for each flow is 1 unit.

The shortest path for flow1 includes the links (13,14). Since flows 1 and 0 are not combinable, if flow1 transmits a message via its shortest path, flow0 is unable to utilize any of its shortest path that contain the link (13,14). Similarly, the shortest path for flow2 comprises the link (19,20). Given that flows 2 and 0 are also non-combinable, flow0 cannot send messages through any of its shortest paths containing link (19,20) while flow2 is active. For flow5, its shortest path consists of the link (18,17), since flows 5 and 3 are non-combinable as well. flow5’s transmission along this route prevents flow3 from using any of its shortest paths containing (18,17). If flow3 transmits its message via the shortest path involving (21,14), then flow0 is again precluded from sending messages via this route because both remaining shortest paths available to it include these same links. Thus far, it has been established that flow3 can only transmit messages through an alternative route: 8-18-21-16-15-5. Notably, one of the two shortest paths for flow0 includes (18,21), and since flow0 and flow3 are not combinable, flow0 can only send messages via the shortest path 0-10-11-20-21-14-4. Therefore, the scheme in which every flow is capable of transmitting messages via its shortest path is unique.

Based on the shortest path method, if a unique shortest path exists, it must be selected. When multiple shortest paths are available, one is randomly chosen as the flow route. Given that the scheme where every flow transmits messages via the shortest path is unique, the probability of successfully scheduling each flow along the shortest path using the shortest path method [11] is calculated as $\frac{1}{6}\times \frac{1}{3}=\frac{1}{18}$.

Based on the two-shortest path method, we calculate the probability that the shortest path for flow0, which includes the link (18,21), is chosen as an alternative path. The total number of combinations for selecting two paths from a set of six paths is given by $C_{6}^{2}$. Among these combinations, there are five combinations that include the link (18,21). Therefore, the probability of selecting a path containing (18,21) as an alternative path can be expressed as $\frac{5}{C_{6}^{2}}=\frac{1}{3}$. Similarly, we assess the probability that the shortest path for flow3, which contains the link (18,17), is selected as an alternative path. In this case, there are two valid combinations out of $C_{3}^{2}$ possibilities. Thus, this probability can be calculated as $\frac{2}{C_{3}^{2}}$=$\frac{2}{3}$. Consequently, using this method, the overall probability that all flows can be successfully scheduled utilizing their respective shortest paths is computed to be $\frac{1}{3}\times \frac{2}{3}=\frac{2}{9}$.

Based on the three-shortest path method, the probability of selecting the shortest path of flow0, which includes the link (18,21), as the alternative path can be calculated as follows. The total number of combinations for selecting three paths from out of six paths is represented by $C_{6}^{3}$. Simultaneously, the number of combinations that include the link (18,21) is denoted by $C_{5}^{2}$. Therefore, the probability of selecting a path containing (18,21) as an alternative path can be expressed as: $\frac{C_{5}^{2}}{C_{6}^{3}}$ = $\frac{1}{2}$. Similarly, for flow3, whose shortest path includes the link (18,17), this specific path is selected as an alternative with a probability of 1. Consequently, under this method, the probability that each flow can be successfully scheduled using one of the shortest paths is 0.5.

**Figure 12:**
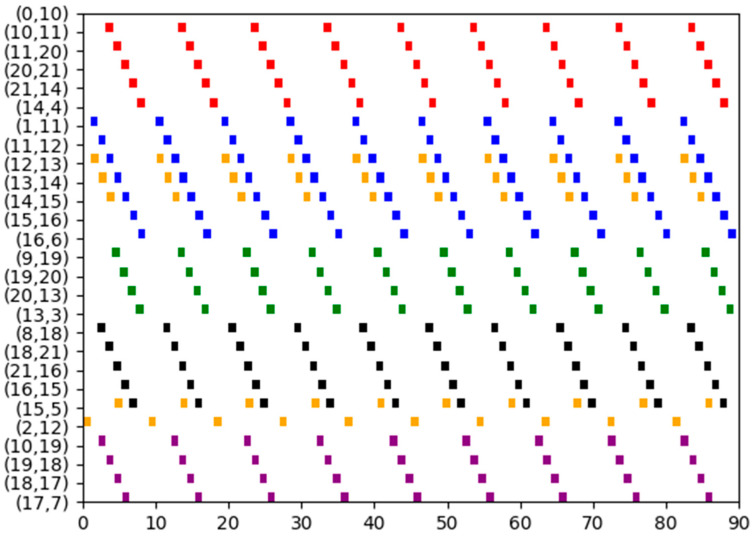
The schedule Gantt chart of Experiment 5. The schedule results of flow 0 to 5 are represented by red, blue, green, black, orange, and purple, respectively.

The proposed method in this paper ensures that each flow is scheduled along the shortest path with a probability of 1. As illustrated in Figure 12, the Gantt chart shows the schedule, where flow0, flow1, flow2, flow3, flow4, and flow5 are represented by red, blue, green, black, orange, and purple, respectively. The SP-ILP method and DA-ILP method could not find a successful schedule in this experiment.

### 5.4. Runtime

Table 6 describes the comparison of the runtime of the GA-DE method proposed in this paper with the SP-ILP, DA-ILP, and JRS-ILP methods in the designed five experiments. It can be seen that with the differences in network scale and traffic characteristics, the SP-ILP method cannot find feasible solutions in Experiment 4 and Experiment 5, and the DA-ILP method cannot find feasible solutions in Experiment 3, Experiment 4, and Experiment 5. The reasons have been analyzed in the previous text. Both the JRS-ILP method and the GA-DE method proposed in this paper can find feasible solutions in all experiments. In terms of the running time of the algorithm, with the expansion of the network scale and the increase in the amount of traffic, the running time of the JRS-ILP method will increase sharply because the number of constraints that need to be solved increases exponentially. However, the GA-DE method proposed in this paper does not have this problem.

### 5.5. Discussions

The routing and scheduling problem for critical flow in time-sensitive networks has consistently been a focal point of research. Existing studies predominantly address the routing and scheduling challenges either by jointly solving them using SMT or ILP solvers, or by first performing pre-routing with techniques such as shortest path or conflict-aware methods, followed by solver-based scheduling. However, as the network size and the number of time-sensitive flows increase, the number of constraints required to be established by SMT and ILP methods grows exponentially, leading to prolonged solution times and, thereby, restricting their practical applicability and scalability. In contrast, while heuristic algorithms like bio-evolutionary algorithms exhibit relatively high computational complexity, their complexity does not escalate dramatically with the expansion of problem scale, thus inherently offering better scalability for addressing routing and scheduling issues. This paper proposes a TSN path selection method based on the genetic algorithm. A fitness function is designed to comprehensively consider factors such as flow combinability, route length, and network load, thereby enabling effective route selection. To further reduce the search space of the genetic algorithm, infeasible paths are eliminated in advance by leveraging the theory of flow combinability analysis. For the scheduling problem, the differential evolution algorithm is employed. By taking the number of constraints that a feasible schedule must satisfy as the objective function, a direct and efficient coding scheme is devised to significantly reduce the algorithm’s search space. Experiments conducted on networks with varying topological architectures and flow characteristics validate the feasibility of the proposed method. However, this method, which simulates biological evolution, relies on continuous iteration to search for feasible solutions, unlike exact solvers that can guarantee optimal solutions. Additionally, when addressing flow routing and scheduling in small-scale networks, the algorithm exhibits high complexity and requires extended solution times.

## 6. Conclusions

In this paper, a novel method based on evolutionary algorithm is introduced for the routing and scheduling of time-triggered flows in TSN. The proposed genetic algorithm approach optimizes routing by leveraging flow combinability to enhance effective flow grouping, thereby improving schedulability. Furthermore, we developed a differential evolution algorithm-based scheduling approach for TSN. The proposed encoding technique effectively reduces the search space. Five experiments were designed to evaluate the performance of proposed method across different scale topologies with various numbers of flows. The results indicate that the proposed method not only enhances the flow along the shortest paths for scheduling, but also significantly reduces execution time in large-scale scheduling scenarios. However, the proposed TSN scheduling method based on the differential evolution algorithm is currently limited to scenarios with zero jitter and no frame loss. In future work, we aim to extend the differential evolution-based scheduling approaches to handle scenarios involving jitter and frame loss.

## Figures and Tables

**Table 1:** Flow parameters of Experiment 1.

	Talker	Listener	Size	Deadline	Period	The Shortest Path
Flow0	1	5	35	150	150	1-6-8-5
Flow1	2	4	24	100	100	2-6-8-4
Flow2	3	4	24	100	100	3-7-8-4

**Table 2:** Flow parameters of Experiment 2.

	Talker	Listener	Size	Deadline	Period	The Shortest Path
Flow0	11	9	24	300	300	11-3-2-1-9
Flow1	9	13	24	300	300	9-1-2-3-4-5-13
Flow2	13	9	24	300	300	13-5-4-3-2-1-9
Flow3	9	8	24	300	300	9-1-0-8
Flow4	13	8	24	300	300	13-5-4-3-2-1-0-8
Flow5	11	10	24	300	300	11-3-2-10
Flow6	8	9	24	300	300	8-0-1-9
Flow7	9	10	24	300	300	9-1-2-10
Flow8	10	12	24	300	300	10-2-3-4-12

**Table 3:** Flow parameters of Experiment 3.

	Talker	Listener	Size	Deadline	Period	The Shortest Path
Flow0	34	31	35	500	500	34-16-15-14-13-31
Flow1	34	28	35	500	500	34-16-15-14-13-12-11-10-28
Flow2	30	31	35	500	500	30-12-13-31
Flow3	21	35	35	500	500	21-3-2-1-0-17-35
Flow4	20	18	35	500	500	20-2-1-0-18
Flow5	18	33	35	500	500	18-0-17-16-15-33
Flow6	31	19	35	500	500	31-13-14-15-16-17-0-1-19
Flow7	18	35	35	500	500	18-0-17-35
Flow8	25	20	35	500	500	25-7-6-5-4-3-2-20
Flow9	31	18	35	500	500	31-13-14-15-16-17-0-18

**Table 4:** Flow parameters of Experiment 4.

	Talker	Listener	Size	Deadline	Period	The Shortest Path
Flow0	1	7	1	8	10	1-13-14-15-19-7
Flow1	2	6	1	9	9	2-14-15-19-18-6
Flow2	4	7	1	8	10	4-16-17-18-19-7
Flow3	3	5	1	9	9	3-15-19-18-17-5
Flow4	0	8	1	8	10	0-12-16-20-8

**Table 5:** Flow parameters of Experiment 5.

	Talker	Listener	Size	Deadline	Period	The Shortest Path
Flow0	0	4	1	10	10	0-10-11-20-21-14-4
						0-10-11-12-13-14-4
						0-10-19-20-21-14-4
						0-10-19-20-13-14-4
						0-10-19-18-21-14-4
						0-10-11-20-13-14-4
Flow1	1	6	1	9	9	1-11-12-13-14-15-16-6
Flow2	9	3	1	9	9	9-19-20-13-3
Flow3	8	5	1	9	9	8-18-21-16-15-5
						8-18-21-14-15-5
						8-18-17-16-15-5
Flow4	2	5	1	9	9	2-12-13-14-15-5
Flow5	0	7	1	10	10	0-10-19-18-17-7

**Table 6:** Comparison of runtime (s) between DE algorithm and ILP based methods.

Instance	GA-DE	SP-ILP [29]	DA-ILP [32]	JRS-ILP [37]
Example1	0.34	1.02	1.36	0.80
Example2	3.19	1.20	1.69	0.94
Example3	3.09	1.58	NA	2.41
Example4	1.16	NA	NA	15.75
Example5	0.94	NA	NA	41

## Data Availability

The data that support the findings of this study are available from the corresponding author upon request. There are no restrictions on data availability.

## Abbreviations

The following abbreviations are used in this manuscript:

TSN	Time-Sensitive Networking
QoS	Quality of Service
SMT	Satisfiability Modulo Theories
ILP	Integer Linear Program
GA	Genetic Algorithm
DE	Differential Evolution
RTRS	Real-Time Routing Scheduler
WCED	Worst-Case End-to-End Delay
JRS	Joint Routing and Scheduling
DoC	Degree of Conflict
EPIC	Efficient Probing Instructed by Conflicts
MGA	Mixed Initial Population Genetic Algorithm
GCD	Greatest Common Divisor
SOW	Sum of Weights
LCM	Least Common Multiple
SP	Shortest Path
DA	Doc Aware

## Appendix A

### Appendix A.1. Proof of Theorem 1

Since the requirement is zero jitter, we have $S_{k+1,t}^{i}=S_{k+1,1}^{i}+\left(t-1\right)T_{i}$, where $2\leq t\leq \frac{HP}{T_{i}}$. Given that the transmission time of the first frame of flow i satisfies the precedence constraint, it holds that $S_{k+1,1}^{i}{\geq S}_{k,1}^{i}+W_{i}$ for any $k\in \{1,2,\ldots ,l_{i}-1\}$. Therefore, it follows that

(A1) $$S_{k+1,1}^{i}+\left(t-1\right)T_{i}\geq S_{k,1}^{i}+W_{i}+\left(t-1\right)T_{i}$$

Since $S_{k,t}^{i}=S_{k,1}^{i}+\left(t-1\right)T_{i}$, we have

(A2) $$S_{k+1,1}^{i}+\left(t-1\right)T_{i}\geq \;S_{k,\;t}^{i}+W_{i}$$

It implies that

(A3) $$S_{k+1,\;t}^{i}\geq S_{k,\;t}^{i}+W_{i}$$

Consequently, the transmission time of the *t*-th frame of flow i also satisfies the precedence constraint.

### Appendix A.2. Proof of Theorem 2

Since $S_{l_{i}-1,t}^{i}=S_{l_{i}-1,1}^{i}+\left(t-1\right)\cdot T_{i}$, therefore, the time required for frame t to be completed at the listener is expressed as follows:

(A4) $$S_{l_{i}-1,1}^{i}+\left(t-1\right)\cdot T_{i}+W_{i}$$

The transmission time $S_{1,t}^{i}$ of frame t at the talker is given by $S_{l_{i}-1,1}^{i}+\left(t-1\right)\cdot T_{i}$. Therefore, the duration between the reception time of frame *t* at the listener and the transmission time of frame t at the talker is calculated by Formula (A5).

(A5) $$S_{l_{i}-1,1}^{i}+\left(t-1\right)\cdot T_{i}+W_{i}-{(S}_{1,1}^{i}+\left(t-1\right)\cdot T_{i})=S_{l_{i}-1,1}^{i}+W_{i}-S_{1,1}^{i}$$

Given that the first frame of flow i satisfies the deadline constraint, it follows that $S_{l_{i}-1,1}^{i}+W_{i}-S_{1,1}^{i}\leq D_{i}$. This implies that Formula (A6) holds true.

(A6) $$S_{l_{i}-1,t}^{i}+W_{i}-S_{1,t}^{i}\leq D_{i}$$

Thus, it can be concluded that the *t*-th frame of flow i also meets the deadline constraint.

### Appendix A.3. Proof of Theorem 3

Let $S_{1,1}^{i}$ denote the transmission time of the first frame at the talker, and $S_{1,\frac{HP}{T_{i}}}^{i}$ represent the transmission time of the last frame at the talker. Given that zero jitter is a requirement, it follows that

(A7) $$S_{1,\frac{HP}{T_{i}}}^{i}=S_{1,1}^{i}+\left(\frac{HP}{T_{i}}-1\right)\cdot T_{i}$$

Let the transmission time of the last frame at node $l_{i}-1$ be denoted as $S_{l_{i}-1,\frac{HP}{T_{i}}}^{i}$. It can be readily established that Formula (A8) holds true:

(A8) $${S_{l_{i}-1,\frac{HP}{T_{i}}}^{i}=S}_{1,1}^{i}+\left(\frac{HP}{T_{i}}-1\right)\cdot +\left(l_{i}-2\right)\cdot W_{i}$$

Thus, we have

$$S_{l_{i}-1,\frac{HP}{T_{i}}}^{i}+W_{i}=S_{1,1}^{i}+\left(\frac{HP}{T_{i}}-1\right)\cdot T_{i}+\left(l_{i}-2\right)\cdot W_{i}+W_{i}$$

(A9) $${=S}_{1,1}^{i}+\left(\frac{HP}{T_{i}}-1\right)\cdot T_{i}+(l_{i}-1)\cdot W_{i}$$

Given that

(A10) $$S_{1,1}^{i}+\left(\frac{HP}{T_{i}}-1\right)\cdot T_{i}+(l_{i}-1)\cdot W_{i}\leq HP$$

According to Formulas (A9) and (A10), it follows that

(A11) $$S_{l_{i}-1,\frac{HP}{T_{i}}}^{i}+W_{i}\leq HP$$

Therefore, flow i meets the period constraint.

### Appendix A.4. Proof of Theorem 4

Let $S_{u}^{i_{1}}$ represent the transmission time of the first frame of flow $i_{1}$ at the talker. Given that the serial number of node u on the path of flow $i_{1}$ is denoted as $k_{1}$, we s have

(A12) $$S_{u}^{i_{1}}=S_{1,1}^{i_{1}}+\left(k_{1}-1\right)\cdot W_{i_{1}}$$

Similarly, it follows that

(A13) $$S_{u}^{i_{2}}=S_{1,1}^{i_{2}}+\left(k_{2}-1\right)\cdot W_{i_{2}}$$

We employ Formulas (A12) and (A13) to substitute $S_{u}^{i_{1}}$ and $S_{u}^{i_{2}}$ in Formula (9), resulting in Formula (10). This indicates that Theorem 4 is valid.

### Appendix A.5. Proof of Theorem 5

(1) According to the precedence constraint and Theorem 1, if a path of a flow has *n* nodes, then, the transmission time of a frame from the second node to the (n−1)-th node must be greater than or equal to the reception time of the preceding node. Consequently, the number of precedence constraints is n−2. Therefore, the number of the precedence constraint for flows $i_{1},i_{2},\cdots ,i_{m}$ that are satisfied is equal to $\sum_{i=1}^{m}\left(n_{i}-2\right)$.

(2) According to Theorem 2, if the interval between the reception time of the first frame of flow i at the listener and the talker does not exceed the deadline, it follows that the intervals between the reception times and the transmission times of all subsequent frames of flow i will also remain within this deadline. Consequently, a single flow is required to satisfy only one constraint; thus, for m flows, a total of m constraints must be satisfied.

(3) According to Theorem 3, the receiving time of the last frame of each flow cannot exceed the hyper-period, so m constraints must also be satisfied on this constraint.

(4) According to resource constraint, for the flow $i_{1}^{j}$, $i_{2}^{j}$, …, $i_{k_{j}}^{j}$ passing through the link ($u_{j}$,$v_{j}$), the number of constraints that must be satisfied is expressed by Formula (A14).

(A14) $$\sum_{x=1}^{k_{j}}\sum_{y=1\wedge x\neq y}^{k_{j}}\frac{lcm(T_{i_{x}^{j}},T_{i_{y}^{j}})}{T_{i_{x}^{j}}}\cdot \frac{lcm(T_{i_{x}^{j}},T_{i_{y}^{j}})}{T_{i_{y}^{j}}}$$

Thus, we have

(A15) $$\sum_{x=1}^{k_{j}}\sum_{y=1\wedge x\neq y}^{k_{j}}\frac{{\left[lcm(T_{i_{x}^{j}},T_{i_{y}^{j}})\right]}^{2}}{T_{i_{x}^{j}}\cdot T_{i_{y}^{j}}}$$

where 1≤j≤h. Therefore, the number of constraints that must be satisfied across h links is as follows:

(A16) $$\sum_{j=1}^{h}\sum_{x=1}^{k_{j}}\sum_{y=1\wedge x\neq y}^{k_{j}}\frac{{\left[lcm(T_{i_{x}^{j}},T_{i_{y}^{j}})\right]}^{2}}{T_{i_{x}^{j}}\cdot T_{i_{y}^{j}}}$$

By integrating the four types of constraints discussed above, the number of constraints that a feasible scheduling solution must satisfy is represented by Formula (11).

### Appendix A.6. Proof of Theorem 6

Let the transmission time of flow $i_{k}$ at the talker be denoted as $S_{i_{k}}$; according to $W_{i_{k}}$, the time at which the first frame is received by the listener is equal to $S_{i_{k}}+W_{i_{k}}\cdot (l_{i_{k}}-1)$. Assume that $S_{i_{k}}>D_{i_{k}}-W_{i_{k}}\cdot (l_{i_{k}}-1)$; then, we have the following inequation:

(A17) $$S_{i_{k}}+W_{i_{k}}\cdot (l_{i_{k}}-1)>D_{i_{k}}-W_{i_{k}}\cdot (l_{i_{k}}-1)+W_{i_{k}}\cdot (l_{i_{k}}-1)$$

It follows that

(A18) $$S_{i_{k}}+W_{i_{k}}\cdot (l_{i_{k}}-1)>D_{i_{k}}$$

It implies that the frame received time is longer than $D_{i_{k}}$. That is, the deadline constraint is violated. Therefore, that value of $S_{i_{k}}$ must not surpass $D_{i_{k}}-W_{i_{k}}\cdot (l_{i_{k}}-1)$.
